# Barriers and enablers that influence the uptake of HIV testing among heterosexual migrants in the Netherlands

**DOI:** 10.1371/journal.pone.0311114

**Published:** 2024-10-09

**Authors:** Veronica Martinez Martinez, Hermen Ormel, Eline L. M. Op de Coul

**Affiliations:** 1 Centre for Infectious Disease Control, National Institute for Public Health and the Environment (RIVM), Bilthoven, the Netherlands; 2 KIT Royal Tropical Institute, Amsterdam, the Netherlands; Centers for Disease Control and Prevention, UNITED STATES OF AMERICA

## Abstract

**Background:**

Heterosexual migrant men and women in the Netherlands often face barriers to accessing health services, including HIV testing, that may lead to late-stage HIV diagnoses. This study explored factors of influence in the usage of HIV testing among heterosexual migrants.

**Methods:**

Qualitative evaluation with semi-structured interviews at the Amsterdam-based AIDS Healthcare Foundation (AHF) Checkpoint and one focus group discussion (FGD) conducted during June-July 2023 with 19 participants: interviews with 12 heterosexual migrants from low- or middle-income countries (LMICs) and FGD (n = 5) and interviews (n = 2) with 7 key informants from the (public) health sector. Recorded interviews were transcribed and thematically analyzed, using the framework of Andersen’s Expanded Behavioral Model of Health Services Use.

**Results:**

In total, 55 themes emerged from the interviews and the FGD. Examples include insufficient availability of information on HIV and testing services, and difficulty in accessing these services (e.g. the barrier of the online appointment system of the Centre for Sexual Health (CSH)). HIV test participants expressed free, rapid testing, no appointment required, and a positive experience during their HIV test as enablers to test in the future. Results from key informants showed that poor health literacy and lack of clarity on the healthcare system’s guidelines were barriers for heterosexual migrants in accessing information on HIV and testing services. It also revealed past initiatives and interventions that were successful in reaching at-risk groups such as the integration of HIV testing into sexually transmitted infection (STI) testing, but that were subsequently discontinued due to financial constraints.

**Conclusion:**

Factors contributing to a low HIV test uptake were participants’ perception of limited accessibility of CSH facilities, insufficient available information on HIV (testing) services, and low perception of HIV risk. Unclear policies on accessing HIV/STI testing services at CSHs, and potential missed opportunities for HIV testing at general practitioners were contributing factors identified by key informants.

## Introduction and study objectives

While much has been accomplished in the past four decades to reduce transmission of Human Immunodeficiency Virus (HIV) infections, it remains a global health concern with over 39 million people affected, of whom 2.3 million people include the World Health Organization (WHO) European Region [1, 2]. In 2020, the Joint United Nations Programme on HIV/AIDS (UNAIDS) set out 95-95-95 global targets (i.e., 95% of people living with HIV (PLHIV) knowing their status, 95% of PLWH receive antiretroviral treatment (ART), and 95% of people receiving ART have viral suppression) to be reached by 2025 [3].

Certain vulnerable groups being affected by HIV, such as heterosexual migrants, require more attention to ensure the UNAIDS’ 95-95-95 targets are reached. Stigma and discrimination continue to play roles in the low uptake of HIV testing. Gender also plays a role, with an overall higher proportion of migrant women testing for HIV compared with migrant men [4]. Incoming migrants, especially those from low- and middle-income countries (LMICs), face financial and social challenges in accessing health(care) services [5]. Their cultural and religious diversity may come with different norms, values and beliefs, which can pose dilemmas when trying to improve access to health services. As a result, they may be more vulnerable to experiencing health inequities, thus creating tailored interventions can be a challenge for healthcare professionals [5, 6]. Exploring HIV attitudes and beliefs among heterosexual migrants is therefore needed; to understand factors influencing the HIV test uptake among this group. This is particularly important in large cities where migrant populations are higher concentrated. Recently, the 2022 *Sevilla Declaration on the Centrality of Communities in Urban HIV Responses*, which focuses on ending HIV transmission in urban settings, help cities to involve communities affected by HIV, and UNAIDS support efforts to expand collaborative rights-based approaches to reduce new HIV infections and ensure health for all [7–9].

About one-third of all migrants that arrive in the Netherlands each year are from outside the EU [10]. Migrants from Morocco, Turkey, Suriname, and the Dutch Caribbean form the largest groups, with diversity in origin now expanded to include those from the Americas, Africa, and Asia [11, 12]. In 2022, there were 393 new HIV diagnoses, of which 42% were acquired through heterosexual contact. The Netherlands currently has 22,102 PLWH in care, of whom 44% are migrants and 47% reside in the main cities [13]. While the 95-95-95 target is almost reached for the Netherlands (94-96-96), improvements can be made, especially given that the continuum of care for ‘other men’ (i.e., heterosexual men) and women was 89-93-95 and 94-95-94 in 2022, respectively [13].

Most migrants in Europe who live with HIV are often infected postmigration, which demonstrates a need for prevention and testing in countries of arrival. Studies conducted in Europe (including the Netherlands) showed that over 50% of heterosexual migrants originating mainly from sub-Saharan Africa (SSA), Latin America, and the Caribbean acquired HIV postmigration within the first five years [14, 15].

Sexual health centers (SHCs) and general practitioners (GPs) are the main providers to test for sexually transmitted infections (STI) and HIV in the Netherlands. STI/HIV testing at SHCs is free of charge for high-risk groups (including “those who originate from an HIV/STI endemic area”) and HIV care is reimbursed through Dutch health insurance [16]. While the Ministry of Health, Welfare and Sport is primarily responsible for health promotion and prevention, the health system is decentralized, with municipalities typically overseeing preventive screenings and long-term outpatient services [17, 18].

At the end of 2022, there were 1390 people who remained undiagnosed for HIV in the Netherlands [13]. Over the past two decades, a trend of low HIV testing rates can be seen among heterosexual migrants in the Netherlands. Migrants from HIV endemic countries residing in the Netherlands often face barriers to accessing healthcare and bear the burden of poverty, unemployment, and lack of education, which can influence their uptake of HIV testing [19–22]. Other factors associated with low test uptake among heterosexual migrants include fear of social exclusion by their communities, low-risk perception and low functional health literacy [21, 23]. Consequently, they are more often diagnosed with late-stage HIV infection (CD4 count below 350 cells/mm^3^) [20]. Late-stage HIV diagnosis requires more attention, taking into consideration region of origin [13]. More focus is also needed on migrant community engagement, financial resources, and political support as outlined in the Sevilla Declaration and the Dutch National Action Plan on STIs, HIV and Sexual Health [7, 24]. The Sevilla Declaration helps cities and municipalities to involve vulnerable communities, and supports UNAIDS efforts to expand collaborative rights-based approaches to reduce new HIV infections. The Dutch action plan includes goals about tailored interventions for subpopulations at higher HIV risk including those with a migration background, such as outreach testing and indicator-based testing at general practices and hospitals.

The COVID-19 government regulations that negatively impacted HIV testing rates in the Netherlands requires active (tailored) case findings of HIV along with strategic scale-up of HIV testing services among heterosexual migrant groups [2, 13, 16].

To contribute to the Declarations to end the HIV epidemic, and reduce inequities in HIV testing service delivery, this study focused on heterosexual migrants in the Netherlands, and aimed to explore factors of influence in the uptake of HIV testing and identify experiences on using HIV testing services. We also aimed to understand the perception of health providers and policy advisors on reasons for late-stage HIV diagnosis among this target population. Recommendations will be formulated to inform policy makers and health providers on strategies for improving the uptake of HIV testing.

## Methodology

### Study design and target population

This qualitative study examined the barriers and enablers of HIV testing usage among heterosexual migrants in the Netherlands. The city of Amsterdam served as the study site being a densely populated area of more than 250,000 migrants [25]. Other city characteristics included various age groups and socioeconomic statuses, a mixture of low-threshold, public, community-based, and private HIV testing facilities, and an overall HIV prevalence higher than 0.5% [13].

The non-governmental organization (NGO) AIDS Healthcare Foundation (AHF) Checkpoint was chosen as the facility to conduct semi-structured interviews (SSI) as it provides a low-threshold, no-cost rapid HIV testing service to everyone, which assisted in finding diverse participants. The SHCs, an additional NGO, and research institution served as the main organizations for key informants, including STI nurses, policy advisors, and GPs. Furthermore, the ministry of health was also approached, but was unable to deliver someone to participate in this study.

Eligible heterosexual migrants met the following criteria (based on AHF Checkpoint’s HIV test assessment form): identified as female or male, were 18 years of age or older at the time of the SSI, were a migrant from an LMIC in Europe, Africa, Asia, the Middle East, or Latin America, and identified as heterosexual. Migrants living in the Netherlands less than five years were considered to be ‘short-term’, while those living in the country five years or more were considered to be ‘long-term’.

The study recruited a total of 19 participants comprised of 12 HIV test clients and 7 key informants from the (public) health sector. Convenience sampling was used to recruit HIV test clients for a SSI on-site at AHF Checkpoint, during the days that the interviewer was present. This was done by communicating the study guidelines to the on-site HIV test counselors to inform and invite eligible clients after their HIV test to participate without influence or pressure. A study invitation poster in English and Spanish was posted in the lobby and HIV testing office. Two study participants, who had never tested for HIV, were recruited through word of mouth by Checkpoint clients who saw the study invitation flyer.

The selection criteria for key informants were that they be either a GP, STI nurse, or policy advisor, be professionally involved with HIV testing among migrants, and have a working knowledge of the English language. Purposeful sampling was used to recruit seven key informants. Key informants were invited for the FGD via email without influence or pressure.

### Data collection and analyses

The principal investigator (PI) conducted interviews with HIV test participants and the FGD with key informants (both face-to-face) based on the Andersen’s Expanded Behavioral Model of Health Service Use conceptual framework, using topic guides [26, 27]. We focused on HIV negative participants given the ethical parameters of the study, and the lesser likelihood of people living with HIV (PLHIV) wanting to be interviewed immediately after their test result.

Two key informants could not attend the FGD and instead suggested submitting their input via a Word document using the FGD topic questions as a guide (further referred to as ‘interviewed key informants’). One interview was performed in Spanish while the rest, including the FGD, were performed in English. All respondents provided informed consent before the interviews and FGD. Permission for the PI to audio record and take written notes of the interviews and FGD was granted by all study participants.

The interviews and FGD were transcribed manually immediately after each session, and the interview performed in Spanish was first transcribed before being translated into English. Anonymity and confidentiality were ensured by not asking participants for any personal identifiers and not sharing any personal information given by key informants (i.e., name, email address, and phone number). Data collection continued until no new topics emerged.

For coding, a hybrid approach was taken, with the research topic guides informing the development of a coding frame matrix, including emerging themes, based on a conceptual framework (for details: see conceptual framework). The data was coded into themes and subthemes from the interviews and FGD. The data was analyzed using this conceptual framework and the latest NVIVO software was used to classify, sort, and arrange the data in order to identify themes and patterns.

The study aimed to reach a diverse group of participants and triangulate data through searching for overlapping themes between the HIV test participants and key informants, and with policy documents to ensure a thorough investigation into the research topic. A number of measures were employed to achieve quality assurance: piloting and adjusting the interview topic guide, accommodating Spanish speakers, audio recording the interviews and FGD, safe data storage techniques (including deletion of interview and FGD audio recordings after the final publication of the scientific paper), and regular follow-ups with the other two researchers, and cross-checks with the last author.

### Conceptual framework

The study was guided by the Andersen’s Expanded Behavioral Model (HBM) of Health Service Use, which was adapted from the Andersen-Newman 1995 Framework of Health Service Utilization [26, 27]. The model asserts that health service use is the result of understanding why and how people use health services, examining disparities in access to healthcare, and contributing to the development of policies related to equitable access to care [28]. Furthermore, population characteristics and environmental characteristics (external or health system) may influence health behavior, which in turn influences health outcomes [26]. The study used population characteristics (i.e., psychosocial, enabling, and need factors) as outcome variables based on the adapted conceptual framework.

Psychosocial factors have an impact on decision-making about planned or intended behavior related to HIV testing, using four domains: knowledge of HIV, attitudes towards HIV testing, social and cultural norms on HIV and testing, and perceived behavioral control over HIV [26].

Enabling factors may influence the frequency of usage of HIV testing services. The lack of availability of appropriate resources and support to access health services and care at the individual, contextual, community/social network, and structural levels may impede care that is necessary due to the low supply of services, or financial means [26]. The ability to access these resources (e.g., low-cost HIV testing) and health facilities are essential [26].

Need is related to how people perceive their own level of health and functionality (including their risk perception of HIV infection) as well as the perception from a GP or HIV test counselor on an individual’s health and needs [26]. The perceived severity of one’s health, access to primary health care and health education, and the availability of funds and/or incentives can all have an impact on one’s perceived need [26].

The expanded Andersen HBM model was used as a framework to analyze the results of this study (deductive approach). Themes that emerged from the interviews and FGD that did not align with this framework were added separately (inductive approach). We focused on the population characteristics and health behavior elements as these were the most relevant in relation to the usage of HIV testing services. Some results sections include information gathered from the key informants that either support or contradict the findings. An overview of Anderson model main domains and emerging themes is presented in S1 Table.

### Ethical clearance and considerations

The KIT Research Ethics Committee (REC) approved the research protocol on 25th of May 2023. Ethical aspects involved collecting verbal informed consent from all participants (audio recorded), interview data anonymously, ensuring confidentiality of participants, and applying participant codes when analyzing and using data in the results section. Furthermore, coding and transcription reliability was checked by a second researcher to ensure the process went according to the study protocol and the REC’s recommendations.

## Results

Out of 12 HIV test (HT) participants, seven resided in the Amsterdam region, and five were from other regions in the Netherlands. Most were highly educated, age range was 20–47 years, duration in the Netherlands varied from 9 months up to 45 years, and several worked in information technology. Regions of origin of the HT participants were Europe, the Middle East, South-East Asia, the Caribbean, Central America, and North and West Africa. Of the 12 HT participants, two had never been tested, six were first-time testers, and four had previously tested between one to four times (Table 1). All key informants had a medical and/or public health background; six out of seven were of Dutch origin (Table 2).

**Table 1:** Characteristics of HIV test participants for interviews.

Participant Code	Age	Gender	Country of Origin	Duration in the Netherlands	Type of Tester
P-1	33	Female	Romania	1,5 years	First-time
P-2	28	Male	Romania	2 years	First-time
P-3	27	Male	Iraq	2 years	Repeat
P-4	22	Male	Iran	1,5 years	Repeat
P-5	35	Male	India	3,5 years	First-time
P-6	27	Male	Turkey	13 years	First-time
P-7	26	Male	Indonesia	21 years	Repeat
P-8	20	Female	Bulgaria	9 months	First-time
P-9	24	Male	Nigeria	2 years	Repeat
P-10	35	Male	Mexico	5 years	First-time
P-11	40	Male	Suriname	30 years	Never-before
P-12	47	Female	Morocco	45 years	Never-before

**Table 2:** Characteristics of key informants for focus group discussion (K-1 to K-5) and interviews (K-6 and K-7).

Participant Code	Job Function	Gender	Organization Type
K-1	Policy Advisor	Female	Research institution
K-2	STI Doctor and Policy Advisor	Female	Public health NGO
K-3	Nurse and Research Coordinator	Female	Public health facility
K-4	Nurse	Female	Public health facility
K-5	General Practitioner	Male	General health practice
K-6	Senior Project Officer	Female	Public health NGO
K-7	Senior Project Officer	Male	Public health NGO

### Psychosocial factors influencing the usage of HIV testing services

The psychosocial factors involved four domains regarding HIV testing: knowledge of HIV, attitudes towards HIV testing, social and cultural norms on HIV and testing, and perceived behavioral control over HIV. The domains originated from the expanded Andersen HBM and all emerging themes (n = 55) came from the interviews and FGD (see S1 Table).

#### Domain: Knowledge of HIV

Knowledge of HIV (including modes of transmission) and HIV test locations were themes that were discussed. While a few HT participants could name at least one HIV transmission mode and one body fluid that can transmit HIV, the overall knowledge of HIV was low. Eight HT participants could name at least ‘blood’ as one body fluid, while two could not name any body fluids that can transmit HIV, and also incorrect examples were given,

*“[…] Of course, if from a pool or urine or if you touch someone else’s blood or something. Um…just blood? I imagine maybe semen, but not sure. Not sure if it’s true or not. I have no idea”*—P-2

Some indicated that they briefly learned about sexual education in primary or high school, but those from Eastern Europe and the Middle East stated that it was not taught in their country of origin. The window period of HIV testing, HIV self-test, treatment for HIV and subsequently living a normal life were emerging themes among three HT participants and some key informants. As HT participant P-1 said,

*“I know that you can get treatment. So, if you have it [HIV], you can just have a normal life. I have a sister-in-law that is HIV positive. But you have a normal life, she just made a baby.”*

Two HT participants understood the need to wait the right amount of time before going for an HIV test (i.e., the window period). One mentioned that he knew to wait at least a couple of months before testing for HIV in order to get a correct result, while the other who had never tested for HIV said,

*“As I know you have to wait. I know that you have to wait three months or something like this.”–P-12*

Online (Google) searches were discussed as the main source of information on HIV and where to find HIV test locations. In addition, four HT participants named the SHC of the public health service (*GGD* in Dutch) as a place they knew where to test for HIV. The GP was mentioned by three others as a known provider for HIV testing. One HT participant was not aware of other HIV test sites, but mentioned HIV self-tests:

*“Well, this one [AHF], but other than this… I remember seeing some websites where you can also like order online. Like some of these rapid testing kits, but then that’s pretty much it.”–P-4*

#### Domain of attitudes towards HIV testing

The attitudes of HT participants consisted of their views on HIV testing and its importance. For half of the HT participants, their visit to AHF Checkpoint was the first time they tested for HIV in their lifetime. The majority of HT participants felt that HIV testing was an important way to prevent the spread of HIV and to not only protect one’s safety, but that of others as well. First-time and repeat testing were emerging themes. While two HT participants had never tested for HIV, they held the attitude that testing is important so that one is aware of whether or not they are transmitting the virus. As one said,

*“It is important for people to do it [testing] because then you’re also aware of whether you’re transmitting or not to other potential candidates, so it’s important to be safe at any time, not only for yourself, but also towards others.”–P-11*

However, another HT participant pointed out,

*“For me not that important, but I can imagine for a lot of other people, and yeah, maybe gay community it is very important. I had never thought about testing for HIV before.”–P-7*

The theme of ‘repeat testing’ emerged among those who had previously tested as being equally as important in prevention of HIV infection with one stating,

*“I think it’s kind of like one of those things that […] people have sex and a lot of people might not know that they might be HIV positive. So, if there was like a culture which advocates for a more repeated testing of HIV, then it prevents it from leading into AIDS and then potentially creating really big consequences for people.”*–*P-4*

Four HT participants were repeat testers (i.e., had tested more than once). One participant, who had tested for the first time in the Netherlands at AHF Checkpoint and three times in their country of origin, confirmed the importance of repeat HIV testing by stating he wanted to make it a routine.

#### Domain of social norms on HIV and testing

Social norms consisted of the following themes: taboo, stigma, cultural perception of HIV, and cultural expectations to care, with sexual orientation as an emerging theme.

The theme of taboo was prominent among most HT participants with many stating that sex remains a taboo subject in their respective cultures. While this was not stated as a deterrent to test for HIV, one participant who had never tested for HIV said,

*“I think looking at myself, that’s [taboo] one of the barriers I had to go through myself—living in the Netherlands—because then you see also the difference is in terms of back home, we barely talk about sex and sexuality and sexually transmitted diseases.”–P-11*

HIV test-related stigma was also mentioned by several HT participants as a result of people’s perception of HIV in their culture. As one stated,

*“People were mocking it [HIV testing], they weren’t taking it seriously. They thought it was useless, […], but I think it’s even a bigger problem about people’s portrayal of HIV because then grows a stigma that is hard to break off.”–P-3*

One HT participant stated that while HIV is talked about, sex is only mentioned in terms of abstinence-only. And, while HIV testing is available in his country, stigma remains:

*“The education system does mostly talk about the illness, but with regards to sex, it’s like mostly to abstain from it. So, people are aware of it. They’re also like free testing. […]. But yeah, it’s like there’s a bit of stigma with it,”–P-4*

As migrants came from different cultural backgrounds, they had varying views on expectations to care. Most stated they were accustomed to going anywhere in their country of origin for health services. As one mentioned,

*“For example, back home, if I register to my GP, I don’t have to be in that area. I can just go to one in another city,”–P-2*

Key informant K-1 added to this by explaining,

*“But still their [migrants] expectancy from the healthcare might be quite different because of their cultural background. So, they go to a doctor, and they expect to get some medication whilst in the Netherlands; GPs are there to kind of advise and counsel. They [migrants] are also disappointed in what they get.”*

Three HT participants mentioned paracetamol as the most common practice by GPs for treatment of general health issues. One key informant (k-5) also acknowledged this, and mentioned that this could result in trust issues with GPs. Another key informant also brought this up as a potential culture shock for migrants, stating,

*“One of the things that really is an issue, is not only the bureaucracy of it all, but also the comment: ‘Start by taking paracetamol.’ This can be a culture shock and can result in care avoiders in the future.”–K-7*

#### Domain of perceived behavioral control over HIV

Perceived behavioral control referred to HT participants’ reasoning behind deciding (not) to previously test for HIV and the time they took to decide to test. There were two main themes: risk perception and behavioral skills, with stable relationship and condom use as emerging themes. Fear, shame, and no sexual partner were mentioned to a lesser extent. Two participants who had never tested for HIV and one first-time tester stated that being in a stable or long-term relationship was their main reason for not testing for HIV. One of the never-before HIV testers stated,

*“If you know somebody for a long time and you had a long-term relationship, then there’s no need to test.”–P-11*

Shame and not having a sexual partner were mentioned by two HT participants, respectively. Fear was also mentioned by participants and some key informants, and mainly came from HT participants not wanting to know their result at the time, with one first-time tester stating he is a hypochondriac.

*“I was afraid. I think fright is by far the biggest concern I had because I’m afraid of a lot of stuff and like I am hypochondriac.”–P-2*

Key informant K-6 further explained in the context of HIV treatment,

*“I think it depends strongly on their country of origin and on the HIV epidemic in their country. People who come from specific African countries where they have experienced the worst aspects of the HIV epidemic fear, are not necessarily familiar with the fact that with a good treatment they can live as long as people without HIV. Therefore, they don’t want to get tested and deny the risks.”*

The majority of HT participants took less than a week to decide to test for HIV from the moment they thought that they should test. For one participant, it took nine months and for another three years before the first HIV test.

### Enabling factors on the usage of HIV testing services

Enabling factors involved three domains: the availability of HIV testing services (primarily in Amsterdam, but also other main cities in the Netherlands), including the accessibility to those services, and the openness to speak about HIV and/or testing.

#### Domain of availability of HIV testing services

Three HT participants came as economic migrants, four came when they were young (age range 2–14) when their families immigrated to the Netherlands. Others came for school or job opportunities). Some HT participants mentioned the availability of HIV testing services to be overall good in the Netherlands. While one participant was only familiar with AHF Checkpoint, he also knew that AHF had testing sites in other cities. As he stated,

*“I only know of this one [AHF Checkpoint] and I know that they go to Rotterdam every Wednesday, so it’s the same organization. They have other locations. Sometimes, I know that they went to Eindhoven a few times. I don’t know of any other organization that does the same.”–P-3*

Themes such as free, without appointment, and rapid testing were seen as the more important determinants to testing at AHF Checkpoint. Other themes mentioned were discreet and convenience in terms of the proximity of AHF Checkpoint and its opening hours. As one stated,

*“I feel like this is the easiest place [AHF Checkpoint] because I came here because I couldn’t find anywhere open today [Saturday]. Or maybe you have to like book appointments, so it’s really convenient and very quick for me.”–P-9*

#### Domain of accessibility to HIV testing services

Easy accessibility to HIV testing services was mentioned by several HT participants. A few encountered some difficulty in accessing HIV testing services prior to finding AHF Checkpoint. One HT participant who is a long-term migrant said,

*“It’s difficult because it’s not very easy…places to find with information [on HIV testing]. Yeah, nobody talks about this. I read many people don’t know they got HIV here in the Netherlands, so they live with HIV and they give to all the others because they don’t know they got it.”–P-6*

One HT participant was glad to have found AHF Checkpoint, as he said,

*“And, basically with the challenge of where I can go without appointment quickly […], because making appointment is very difficult here in Amsterdam.”–P-5*

The SHCs at the GGDs provide testing services to those residing in their municipality and who meet certain requirements such as age and sexual preference, as stated by key informants. HT participants mentioned AHF Checkpoints having no such restrictions and people from other postal code areas are also allowed to test for HIV. Regarding the age requirement, one HT participant said,

*“It has been pretty bad actually with the GGD because I remember that I wanted to [test] there because friends told me about that you could get free STD test until your 25*^*th*^*. After your 25*^*th*^ *you have to start paying for it.”–P-7*

Regarding the postal code requirement, another participant stated,

“*For example, if I am here, because I live in Haarlem. But if I want to search in the GGD of Amsterdam it’s like at the time of putting in the post code and your post code is from Haarlem, you can do absolutely nothing in Amsterdam, and they close the questionnaire and send you to Haarlem.”–P-10*

While none of the participants stated language being a barrier when trying to access HIV testing services at the SHC or GP, key informant K-3 discussed how the SHC provided an online system to make appointments, but encountered some challenges:

*“Actually, we changed our system for making an appointment to an online one. And, by doing it we saw that we were missing persons who were not Dutch-speaking and not online literate enough. But I think the online appointment is an extra handicap because you just cannot phone and make an appointment or ask questions. You have to fill out a form online. And that’s too hard. I think even if you’re Dutch and not very literate online, that’s really a problem.”–K-3*

#### Domain of openness to speak about HIV and/or testing

This domain referred to whether or not HT participants were able to bring up the subject of HIV and/or testing. There were three themes: family, friends, and colleagues.

While a couple of HT participants felt it would be fine to approach their family about the topic of HIV (testing) and that they would feel supported, most felt that it would not be a good idea. As one participant discussed,

*“I think with my friends it would be fine, but I think if it’s with family then it’s a bit taboo because there’s a lot of misinformation about the way it’s transmitted and how it first started and who in particular is it more common with, then all these assumptions. It wouldn’t be a good topic to bring up.”–P-3*

Despite several HT participants coming from conservative cultural backgrounds, the majority felt that they could speak openly with their friends. However, one participant who had never tested for HIV pointed out with regards to family and friends,

*“The topic is no problem, I think—to talk about HIV. But if I have to do a test, then it will be another thing. Then it is ‘did you sleep with somebody else?’, ‘did your husband sleep with somebody else?’, ‘why should you take a test?’ I think that would be if I told somebody from my background.”–P-12*

All HT participants said they would not discuss HIV and/or testing with their colleagues due to either feeling uncomfortable or unnecessary to do so. However, one mentioned,

*“I think there’s other STD’s that are more likely to be brought up during discussion, but not HIV because where I work it’s not a branch where people talk about HIV. Maybe about other STD’s. But honestly, I’ve never talked to my colleagues about it.”–P-7*

### Need factors influencing the usage of HIV testing services

The need factors consisted of two domains: perceived need (HT participant) and evaluated need (Key informants). Perceived need had the theme of an HT participant’s perception of HIV risk, while evaluated need had the theme of key informants’ (GPs’) beliefs on heterosexual migrants’ risk for HIV.

#### Domain of client’s perceived need for HIV test

An HT participant’s perceived risk for HIV influenced their decision to test, with unprotected sex (mostly from a one-night stand or a new partner) and physical symptoms being an emerging theme. One HT participant mentioned injecting drug use:

*“I mean, if I’m not doing any sexual interaction with any other partners, and I don’t have any drugs addiction. […]. I mean, it’s not always it should happen with only sexual intercourse. For example, if we are exchanging some drugs and all the blood exchange or something like this, the injection.”–P-5*

#### Domain of evaluated need for HIV test

Since most HT participants had either tested for the first time in their lifetime at AHF Checkpoint or had never been tested for HIV, most had not been to a GP for an HIV test. Two HT participants, both long-term migrants, had been to a GP before to test for HIV. However, one of them who had a first HIV test at AHF Checkpoint discussed how their GP turned them away due to their low risk:

*“My doctor says it’s not necessary because it’s very low risk with Dutch girls. Only if I slept with girls from other countries. He told me that. He says not necessary for me now, because you don’t sleep with [girls] in the Red [Light] districts, it’s just not necessary.”–P-6*

To further illustrate this point, key informant K-7 pointed out,

*“Healthcare professionals have multiple reasons not to test for HIV. For example, they don’t want to come across as stigmatizing because their client is from a certain country. But sometimes healthcare professionals don’t test for HIV because their client isn’t MSM.”*

### Use of health services (i.e., HIV testing services)

The usage of HIV testing services consisted of three main themes: expectations of the HIV test service, experience during the HIV test, and competency of the health providers (i.e., HIV test counselor(s)).

Having a knowledgeable HIV test counselor, safe space, anonymity, no judgement, reliability of test, friendly staff, and relaxing setting were mentioned among the HT participants as expectations they had from AHF Checkpoint, with one explaining,

*“Answering all the questions that people might have, because in some cases people have a lot of fear towards this thing [HIV testing]. Even if, like you were positive, that it’s fine. It’s just like any other virus; you can contain it. But yeah, also providing a safe space.”–P-3*

Another added,

*“I think the fact that I don’t feel judged. Let me know that I am in a safe place. That they are not going to make any judgment or something that could affect me negatively.”–P-10*

None of the HT participants who had previously tested ever had a negative experience during an HIV test. Participants were asked what they would do or what their reaction would be if they were to ever have an HIV testing experience that was negative or uncomfortable. Some stated that in that case, they would still return to test for HIV for health reasons (i.e., wanting to know the result) and because it’s free. Some mentioned they would either ask for someone else at the Checkpoint to test them or go elsewhere. One HT participant was direct and said,

*“Then I don’t want to do with the testing. Yeah, I go to my doctor then.”–P-6*

The 10 HT participants who were recruited after their HIV test at AHF Checkpoint expressed that they had a positive experience during their HIV test (two others were never-before testers). Those who experienced some anxiety or panic prior to taking the test mentioned that the HIV test counselor made them feel relaxed and at ease. However, one participant compared his experience to that of their country of origin when entering the Kilo Store (i.e., entrance of the AHF Checkpoint):

*“It’s much different from here [the Netherlands], you know, even just going for an HIV test. The eyes you get from people around even coming up the stairs to this place [AHF], like people in the store downstairs, they be looking like ‘where you going?’ They be staring, you know like ‘what you doing?”–P-9.*

All HT participants stated that the HIV test counselor on duty had a strong level of competency: They named important attributes that the HIV test counselor(s) possessed: knowledgeable of HIV, reassurance, ability to handle anxiety, and ability to explain the HIV test procedure well. One of the two participants who had never tested for HIV said she would expect the person performing the test to be nonjudgmental, and the other never-before HIV tester mentioned,

*“If that person is open and able to have good conversation skills and be able to relax with you. Help to have a good talk, a good chat…those are all the attributes that add up.”–P-11*

### Dutch healthcare system

This factor consisted of two themes: the Dutch healthcare system in relation to HIV testing (as well as general health services) and discrimination. Health literacy was an emerging theme.

The majority of HT participants claimed to not know very much about the Dutch healthcare system. Three participants stated knowing that one must first register with a GP before obtaining any type of health service (including HIV testing), and that the SHC (GGD) also offers HIV testing. One HT participant expressed, however, that he already expected the SHC to not be a useful resource for him and his partner for HIV testing:

*“So, we already knew it was going to be like ok, let’s review what options the GGD offers and after GGD it’s like, ok well, we saw that the GGD does not work, let’s look for other options.” P-10*

Another HT participant knew of a private clinic in Utrecht, but the cost was a barrier as he said,

*“I know this place [AHF Checkpoint] yeah, and [the clinic] in Utrecht—it’s fast test, 14 days [after risk] it says, and they want 300 euro. But I don’t do that, too much money.”–P-6*

One key informant mentioned that the health system in the Netherlands is decentralized, with GGD facilities serving as additional resources to the regular healthcare system. Regarding the responsibility to offer HIV testing and prevention services, she added,

*“But it’s not a governmental task. That’s exactly the point. That’s the decentralized system we have in the Netherlands and that means that this task is at the GGDs. They are the ones that have to address preventive measures towards the people within their region and they can issue leaflets or whatever in any language you want if you have the finances for that”–K-1.*

Moreover, this key informant added to the complexity of the healthcare system in terms of STI/HIV testing:

*“But it’s a complicated system […], it’s not clear what is paid for, what is not, […] who has access to it.”*

Also, various key informants stated poor health literacy among migrants being the biggest problem because it impedes their ability to know how and where to go for health services. As one key informant stated,

*“I think health literacy in general is the biggest problem because they [migrants] don’t know where to go or have the knowledge that it [HIV testing] might be useful,”–K-2.*

All HT participants stated they did not experience discrimination during their HIV test consultation at AHF Checkpoint or when acquiring general health services in the Netherlands.

### External environment

External environment and its influence on the use of HIV testing services referred to two themes: place of residence, and health policies and guidelines.

Most HT participants resided in the Amsterdam region, but five resided in different regions, of whom three in cities more than 30 kilometers from Amsterdam. Despite those who reside far away still coming to test for HIV at AHF Checkpoint, one key informant stated geographical availability as a barrier to HIV testing.

Regarding HIV prevention awareness geared towards migrant groups, one key informant stated,

*“There’s hardly anything there because that’s not the policy of the ministry. […] We do want to address for instance condom use, so we want to make a campaign that’s broader for a lot of the public. Advertising the use of condoms. But they are absolutely directed towards the general public. There is no emphasis towards migrants at all.”–K-1*

The National Institute for Public Health and the Environment (RIVM in Dutch) guideline regarding which groups to include for STI/HIV testing at SHCs, on which financial payment is based, includes people from an endemic HIV country for HIV testing. In reality, however, it is not guaranteed that these individuals could test for HIV at their local SHC, as SHCs have some liberty to make their own priorities due to financial constraints. As key informant K-1 explains,

*“They [GGD] will prioritize those who are warned for an STI or who have symptoms of an STI. So, just being young or just being from an endemic country doesn’t give you access.”*

One key informant added that a full STI screening may not always be offered even if the SHC is not at full capacity. Individuals wanting further testing will be referred to their GP. HIV testing guidelines for GPs include an indicator-condition approach as well as offering routine testing to at-risk groups. However, missed HIV testing opportunities at GPs still exist, according to key informant K-3.

One key informant explained how her team addressed to the ministry the importance of integrating sexual health at the national level so that cities will integrate it into their local policy. She further discussed that the general attitude of municipalities does not make it easy to find additional financing. Key informant K-3 added,

*“And that’s why we are always very happy that we have the finance only partly from the municipality and the larger part is governmental.”*

One key informant said the SHCs ask for one’s postal code before making an appointment, which she considered a barrier. Another key informant added,

*“But they [migrants] don’t have to show proof. So, I mean they [SHC] can never check if they’re really living there. So, you can call to Haarlem, and you say you’re from Haarlem and you might be accepted at the Public Health Service [SHC], and they won’t ask for proof,”–K-4.*

When K-4 was asked whether or not people knew of this fact, she replied,

*“No, they don’t know.”*

### Perceived health status

The perceived health status factor referred to HT participant’s perception of their overall health and the influence it has on seeking health services, including HIV testing. If participants had a perceived overall good health status, they were less likely to go to the doctor for any health service. In some cases, sexual behavior was the main reason to visit the doctor. As participant P-9 stated,

*“Yeah sometimes you know, you have maybe a flu, and it takes longer to go away and it’s like, ‘why is it taking so long?’ You know, stuff like that. But mainly it’s personally just because of sex.”*

One of the participants who had never tested for HIV said that if she experienced symptoms of any kind, she would go to the doctor, but would not think it was HIV:

*“If I have symptoms, I don’t know if I think about HIV. It’s not the first thing I think ‘Oh, maybe I have HIV. If I had headache or I think my blood pressure is low then I will go to the doctor, but I wouldn’t think about HIV at this moment.”–P-12*

### Evaluated health status

The evaluated health status referred to HT participants’ health being assessed by a health professional (i.e., GP or nurse), in relation to going for an HIV test. Only one participant who had tested for HIV with a GP in the Netherlands discussed,

*“Yeah, but I don’t go there with the idea I want to do an HIV test. I just go there with things that I feel maybe are bad and then they said, ‘you know, just to be sure, let’s do an HIV test. It’s not that I went there because I wanted to take one. Since I was already there they brought it up because of the symptoms I was having, to make sure.”–P-7*

Three key informants mentioned there being a lack of HIV testing even with the introduction of indicator-condition guided HIV testing due to its broad nature and GPs being uncertain of when to offer the test. Another key informant (K-5) said,

*“So, in the general GP training, there’s no HIV specific curriculum, but we of course have training in STDs but it’s pretty superficial. As GP’s we try to assess ‘risk behavior’ and give our patients some education about sexual behavior and its risks. STD diagnostics are offered according to (risky) behavior and/or patients’ wishes. However, we do not outreach to all our patients.”*

Key informant K-2 mentioned the efforts in trying to remedy this,

*“Soa Aids Nederland has been trying to train GPs, especially in Amsterdam but also outside, in diagnostic testing, which is about you’re not going to find HIV, you’re going to be sure there is no HIV.”*

### Suggestions from HT participants and key informants

HT participants and key informants were asked about their suggestions on how to improve HIV testing in the Netherlands among heterosexual migrants. Some HT participants brought up the importance of awareness campaigns, outreach activities, and promotion and normalization of HIV testing (services). All key informants highlighted the need to improve the health literacy of heterosexual migrant groups, particularly men since they tend to enter into HIV care at a later stage due to the difficulty of finding their way into the healthcare system. Many HT participants felt the need to have more free testing services to remove financial barriers. One participant suggested having more HIV testing services available on Saturdays. Also, several HT participants mentioned not having enough available information regarding where to test for HIV. As one pointed out,

*“I do not feel that there is as much promotion of ’come get tested’ or ‘come to these places’. I mean, I see more things like, I don’t know, do your taxes, you know, things like this. It’s not like we enter the country and the first thing they tell you is ah, you have just arrived, take all your things, this is your welcome kit, no.”–P-10*

A key informant corroborated this by saying,

*“The GP is there for people who expect they have a problem, but a lot of migrants wouldn’t know they had risk behavior or that they are at risk of contracting HIV. […] I mean, for somebody who’s entering the Netherlands as an adult, there is not a single moment in their life that they will be getting any information apart from the Internet but also then you will have to have an incentive to be looking for information, and if you don’t have that incentive then you’re lost.”–K-1*

A couple of key informants mentioned past interventions that were successful in reaching at-risk groups such as the HIV Transmission Elimination Amsterdam (H-TEAM), and the integration of HIV testing into other health services (e.g., prenatal screening and HIV/STI testing). However, a few key informants mentioned that the integration of HIV testing into STI testing encountered financing issues. See S1 Data for the final dataset of used and unused quotes of Anderson model main domains.

## Discussion

This study identified factors that contributed to a low uptake of HIV testing services among heterosexual migrants in the Netherlands, namely HT participants’ low knowledge of HIV (including where to test), perception of limited accessibility of SHC facilities, insufficient available information on HIV (testing) services, and low perception of HIV risk. Unclear policies and guidelines on accessing HIV/STI testing services at SHCs and missed opportunities for HIV testing at GPs were contributing factors identified by key informants.

Low overall knowledge of HIV (including transmission routes and where to test) was present among some HT participants, despite interviews having been conducted after the participants’ HIV test counseling session (with the exception of two persons who had never tested). Despite this, the majority of HT participants believed that HIV testing was of high importance. Furthermore, long-term migrants were not very familiar with the Dutch healthcare system, even less in relation to HIV testing services, despite four being raised or living in the Netherlands since their youth.

Psychosocial, enabling, and need factors influenced the use of HIV testing services (including repeat testing). The main reasons HT participants tested at AHF Checkpoint were because they provide free, rapid testing, with no appointment required. The cost of HIV testing was seen as a barrier or enabler depending on the context. The free HIV testing service of AHF Checkpoint was an enabling factor that many participants stated when searching for an HIV test. Conversely, some HT participants and key informants mentioned costs at private clinics and GPs being a deterrent to testing for HIV. While an online HIV self-test was mentioned by an HT participant as an alternative to the high costs at private clinics, it was felt that the cost would still be a deterrent for most migrants.

The postal code requirement and the SHC’s online appointment system were perceived as barriers by both HT participants and key informants. Key informants explained the challenges that migrants face regarding accessibility to and availability of test services. Interestingly, the participants who had never tested for HIV perceived the accessibility to HIV testing services to be overall good, but also felt that most migrants they know are completely unaware of where to test and of the requirements. Any information on HIV and testing services was mainly accessed on the internet with unavailability of information in multiple languages. Moreover, HT participants came from different countries with varying cultures, and thus had different expectations of care. However, this was more reflected in acquisition of general health services.

The issues of accessibility remain a challenge in the Netherlands despite its major cities committed to translating HIV and testing information into multiple languages, and improving the access to this information that goes beyond the conventional methods used by public health institutions, as outlined by the Sevilla Declaration [7]. This prompts more attention being needed on outreaching to community-based organizations who are the leaders in providing services and support to vulnerable affected communities [7]. Furthermore, additional resources and implementation are needed towards improving indicator-condition guided HIV testing to avoid late-stage HIV diagnoses since it has been shown to be efficient in identifying newly diagnosed people [29]. Slight improvements have been made in GP-initiated HIV testing in the Netherlands. However, missed opportunities remain, as illustrated in our study and other studies [30–33]. Insufficient STI/HIV training and mainly having patients initiate the conversation around HIV testing was mentioned by key informants and HT participants.

HT participants perceived their risk of HIV to be low, which led some to feel that an HIV test was previously unnecessary and others to feel that future HIV testing is needed in cases of unprotected sex. HIV testing was also considered more important for the gay community. However, as mentioned by a key informant, heterosexual men may not consider themselves gay or bisexual despite sexual interactions with other men. Also, some participants perceived condom use and being in a stable relationship as reasons to not test for HIV. As studies in the Netherlands have shown, low risk perception of HIV was more likely among heterosexual migrant men and women, and health providers do not always think about testing for HIV in the presence of HIV indicator conditions when patients are of older age [34–36]. Among participants in our study, the majority came from countries where HIV has a strong presence and where it is widely discussed even if not always in the context of sex (education). Moreover, finding the underserved heterosexual migrant (sub)groups remains a challenge. Additionally, health providers continue to face cultural and language barriers that make it challenging to help the migrants that they do reach, illustrating the need for training, and resources.

Literature on never-before HIV tested heterosexual non-Western migrants in Europe showed the main barriers to testing as being the belief of migrants not being HIV infected and the lack of knowledge of HIV, including where to test (for no or low cost) [37–39]. This was partially the case in the two never-before HIV testers in our study who did not feel they were at risk for HIV. However, despite knowing where to test for HIV, they had less knowledge of the topic.

Inter(national) studies have shown that fear of the test result plays a big role in not seeking testing due to perceived consequences of a diagnosis, which include social stigma from communities discovering their result [31, 35, 40]. Only a few HT participants mentioned fear as their reason for not having previously tested for HIV, but many said that in their respective cultures fear does play a major role in people not wanting to test for HIV. Fear may have played a smaller role in our study, due to the recruitment location at an HIV test site. Few HT participants referred to treatment for HIV and stated that one can live a “normal” life on treatment, but none commented on whether or not this knowledge would reduce their fear of HIV. One HT participant was aware that PLWH can give birth to healthy children, which could indicate a need to further educate heterosexual migrant groups on how the life expectancy of PLWH on ART treatment is similar to those living without HIV, and the U = U concept (Undetectable = Untransmissible); a topic that did not come up during the interviews or FGD [9].

While no participants in our study experienced discrimination or stigmatization when trying to access health services (including HIV testing) in the Netherlands, other recent Dutch studies showed the contrary [20, 41]. This could be due to the client-centered approach at the AHF testing location, or due to gender differences given that MSM migrants and migrant women were the ones who experienced discrimination in one study and in the other, the focus was on specific migrant groups (i.e., Turkish-Dutch) [41]. In our study, most HT participants were heterosexual male migrants from various ethnic backgrounds. Also, all participants were HIV negative and in general PLWH have greater chances to experience negative attitudes in their social environment and health care system.

A successful initiative to reduce HIV transmission has been the Amsterdam H-TEAM, which uses biomedical, behavioral, and structural interventions to reach MSM and people who originate from countries with a high HIV prevalence [42]. However, more MSM are reached than heterosexual migrant groups, as mentioned by key informants. A study in Sweden that focused on Syrian and Iraqi migrants saw that interventions with “language-adapted information” about sexual health and testing services improved accessibility [43]. Additionally, outreach activities are especially vital in reaching migrant subgroups that may be more at risk [43]. This intervention could be applied to the Netherlands given the lack of HIV (testing) information in multiple and culturally adapted languages, and should be financially feasible.

### Strengths and limitations

The theoretic (expanded) framework used in this study guided the analysis on the issues raised. The hybrid analysis-approach (deductive and inductive) helped to create and find themes that were not explicitly laid out in the framework. The adaptability of the framework makes it useful in future health service utilization studies, but including the ‘predisposing characteristics’ from the earlier framework alongside the psychosocial factors instead of replacing it is recommended since it would capture more of the e.g., gender, race, socioeconomic and family size issues related to the usage of HIV testing services. In this study, the factors ‘Consumer Satisfaction’ and ‘Personal Health Practices’ were excluded since the former was already reflected in the section on ‘Experience during HIV Test.’ The latter factor was excluded since there was no difference seen between individuals with a healthy or not so healthy lifestyle linked to (ever) testing for HIV.

Furthermore, the successful inclusion of heterosexual male participants in this study was also a strength that provided new insight into the (assumed) lesser willingness of heterosexual migrant men to test for HIV. Despite the HIV test counselors on duty being female (with the exception of a male test counselor on one day of data collection), a larger willingness to participate was among males. Data collection from both HT participants and key informants allowed for confirmation of results (data triangulation). We included two people who had never before been tested for HIV and six first-time testers. Different perspectives were obtained through the recruitment of participants from countries with various cultures and religions. Additionally, a variety of key informants from different work backgrounds provided a well-rounded discussion on reasons for late-stage HIV diagnosis among heterosexual migrants. Two key informants, however, were unable to participate in the FGD and provided responses on our questions on paper. Consequently, these key informants were not able to share and exchange experiences with other key informants, which is a limitation. Having a diverse group of HT participants may have also had some limitations, e.g., not always having males and females within each migrant group. Furthermore, convenience sampling in one location in Amsterdam may have affected representativeness of the study, especially since participants from rural areas were not included, potential issues (e.g., distance to SHCs) that were found in Dutch studies did not play a role in our study [44], and may have affected generalizability of our results. Finally, the challenge of finding participants who had never been tested for HIV was a limitation. The recruitment of participants was in a setting where people already came for HIV testing, which may have caused selection bias and social desirability bias. Participants who were eligible were not turned away even if they had tested previously despite the study protocol looking in particular (but not exclusively) for first-time testers. Only two participants who never tested were included. It remains unclear how this may have affected our results. There was limited literature on never-before HIV testers in Europe, and what was available mainly focused on migrants from sub-Saharan Africa [38, 39].

While language was not presented by HT participants as a major barrier, it does not mean that this barrier does not exist. The selection criteria tool was only available in English and Spanish. As a result, there was potential selection bias with migrants either speaking English or Spanish. Furthermore, given the heterogeneity of migrant populations, they cannot be seen as one cultural group, which limited the study in focusing on specific migrant sub-groups.

Similarly, factors like poverty, employment, and lack of education remain issues in accessing HIV information and testing services, but our participants were relatively well-educated. Moreover, our study was unable to delve into the political aspect of health system financing (except for some statements about a broader involvement of the municipality in providing additional finance for testing) and its influences on the use of health facilities and health providers in providing HIV information and testing services.

## Conclusion and recommendations

This study found several factors that pose a barrier to HIV testing among heterosexual migrants in the Netherlands. Psychosocial, enabling, and need factors are key in understanding why and how migrants use HIV testing services, but the inclusion of other components (of the framework) is also crucial in helping to assess inequalities in accessing these services. AHF Checkpoint was considered by HT participants to be a convenient and easily accessible HIV testing facility. Recognizing the importance of all components can guide policy makers in using evidence-informed interventions to improve the access to and availability of HIV information and testing services in the Netherlands.

The input received from HT participants and key informants helped create recommendations (Tables 3 and 4) which are geared towards policy makers, health providers, and further research development to improve accessibility to HIV testing services among heterosexual migrants and the promotion of these services. Further research is needed on heterosexual migrant groups who have never before tested for HIV, those living in more rural areas, and the factors related to HIV testing usage, including subgroups, to propose tailored interventions that are more effective and meet the specific needs of different cultural groups. Engaging with migrant (sub)groups in an effort to understand their main health concern(s) can help relevant stakeholders create culturally appropriate messages and innovative service delivery programs to better integrate sexual health and HIV testing into other health services.

**Table 3:** Recommendations for policy makers.

**Integrate health literacy and knowledge of HIV testing facilities**	Health literacy and knowledge of HIV testing facilities should be part of the integration process for new arrivals, for instance, by developing an app that newcoming LMIC migrants can use to find general and health services in the Netherlands. The app and/or HIV (testing) information can be provided at first points of entry such as the municipalities when people register for their citizen service number (*BSN* in Dutch) or during their first visit at the GP. Outreach activities can be done throughout neighborhoods with a higher concentration of migrant populations to deliver culturally- and language-adapted HIV information, using staff or volunteers from community-based organizations who themselves are migrants.
**Improve GP-initiated HIV testing**	To improve GP-initiated testing, NGOs such as Soa Aids Nederland and Doctors of the World can collaborate with migrant community organizations that work specifically with PLWH to better train GPs on how to best bring up HIV testing among various migrant populations.
**Expand low-threshold HIV testing services to improve accessibility**	Existing health organizations and facilities (e.g., SHCs, GP) can expand low-threshold HIV testing services by having (no or low cost) availability on Saturdays twice a month with the help of staff or trained volunteer HIV test counselors. These services can also be offered through discreet mobile units (i.e., no HIV-related insignia) going to rural areas where access to SHCs is a barrier.

**Table 4:** Recommendations for health providers.

**Loosen or remove the postal code requirement**	The postal code requirement laid out by the SHCs makes it difficult to reach migrants who may be at risk for HIV infection. The SHCs can loosen or remove this restriction and make it clear to the public on their website to allow people to test for HIV at a SHC outside their city of residence.
**Improve online appointment system (SHCs)**	The SHC can improve their online appointment system by providing the option to call for an appointment to accommodate those who may be online illiterate, and having it available in more commonly spoken languages.
**Make information on HIV and testing services available in multiple languages (most commonly spoken)**	Health facilities and organizations (including SHC, hospitals, and AHF Checkpoint) can make information on HIV and testing available in multiple languages through either allocating funding for translation services or seeking volunteer translators.
**Normalize HIV and testing**	Health providers can increase the knowledge of HIV and testing through neighborhood campaigns and/or combine HIV testing with other health tests that are non-stigmatizing (e.g., cholesterol, iron, glucose, etc.) during primary care visits to embed it as a routine.
**Bundle initiatives with other health providers**	Health providers (including GPs, nurses, and AHF Checkpoint) can learn from each other’s interventions and bundle initiatives through collaboration on HIV testing projects to reach more heterosexual migrant groups.

## Supporting information

S1 Table Overview of Anderson model main domains and emerging themes. (DOCX)

S1 Data Final dataset (used and unused quotes of Anderson model main domains). (DOCX)
