# Supplementary material for: Barriers and enablers that influence the uptake of HIV testing among heterosexual migrants in the Netherlands
Source: PLoS One. 2024 Oct 9;19(10):e0311114. doi: 10.1371/journal.pone.0311114 (PMC11463776; doi:10.1371/journal.pone.0311114)
Supplement: S1 Table — (DOCX) [file pone.0311114.s001.docx]

| **Population**  **Characteristics** | | **Factors** | **Domains** | **Main themes** | **Emerging themes** |
| --- | --- | --- | --- | --- | --- |
|  |  | Psychosocial Factors Influencing the Usage of HIV Testing Services | Knowledge of HIV | • Acquisition of HIV  • Knowledge of HIV testing locations  • Sources of HIV information | • Amount of available HIV information  • Window period  • HIV self-test  • Treatment of HIV |
|  |  |  | Attitudes towards HIV Testing | • Views on HIV testing  • Importance of HIV testing | • First-time testing  • Repeat testing  • Status awareness  • Gay community  • Normalize HIV and testing |
|  |  |  | Social Norms on HIV and Testing | • Taboo  • Stigma  • Cultural perception of HIV  • Cultural expectations to care | • Sexual orientation  • Taboo of sex  • HIV test-related stigma  • Paracetamol  • Trust issues |
|  |  |  | Perceived Behavioral Control over HIV | • Reasons for no previous HIV testing  • Decision to test for HIV  • Risk perception  • Behavioral skills | • Stable relationship  • Condom use  • Fear  • Shame  • No sexual partner |
|  |  | Enabling Factors on the Usage of HIV Testing Services | Availability of Testing Services | • Amount of HIV test locations | • No cost  • Without appointment  • Rapid testing  • Discreet  • Convenience  • HIV (testing) information in more languages |
|  |  |  | Accessibility to HIV Testing Services | • Access to HIV test services (easy/difficult)  • Access to HIV test services (GP or GGD) | • Finding available appointment at GGD  • Postal code  • Age requirement  • Sexual preference  • Online system  • Low-threshold testing  • Outreach to deliver HIV information |
|  |  |  | Openness to talk about HIV and/or testing | • Family  • Friends  • Colleagues |  |
|  |  | Need for HIV Test | Client’s Perceived Need for HIV Test | • HT participant’s HIV risk perception | • Unprotected sex (one-night stand or new partner)  • Physical symptoms  • Injection drug use |
|  |  |  | Evaluated Need for HIV Test | • Key informants’ beliefs on HT participant risk for HIV | • GP’s beliefs on risk perception |
| **Health Behavior** | | Use of Health Services | HIV Testing Services | • Expectations of HIV test  • Experience during HIV test  • Competence of Health provider (i.e., HIV test counselor) | • Knowledgeable HIV test counselor  • Safe space  • Anonymity  • Reliability of test  • Friendly staff  • Relaxing setting  • Non-judgmental |
| **Environment** | | Health Care System | Dutch Healthcare System | • Knowledge of Dutch healthcare system regarding HIV testing  • Discrimination | • Health literacy  • High cost at private clinics  • Decentralized system |
|  |  | External Environment | Influence of External Environment on the Use of HIV Testing Services | • Place of residence  • Health policies and guidelines | • Geographical availability  • Ministry of Health policy  • Financial constraints  • GP-initiated testing  • GGD guidelines |
| **Outcomes** | Perceived Health Status | | HT Participant’s Perception of Overall Health | • HT participant’s perception of overall health and the influence it has on seeking health services (including HIV testing) | • Sexual behavior  • Symptoms |
|  | Evaluated Health Status | | HT Participant’s Health being Assessed by Health Professional at Medical Facility | • HT participant’s health being assessed by GP or nurse | • Indicator-condition approach  • Training GPs |
