# Supplementary material for: Barriers and enablers that influence the uptake of HIV testing among heterosexual migrants in the Netherlands
Source: PLoS One. 2024 Oct 9;19(10):e0311114. doi: 10.1371/journal.pone.0311114 (PMC11463776; doi:10.1371/journal.pone.0311114)
Supplement: S1 Data — (DOCX) [file pone.0311114.s002.docx]

# Quotes associated with the Andersen Expanded Behavioral Model Main Domains

**Knowledge of HIV**

P-1: “Sexual transmission. Like blood. And yes, the cum, or you can get stinged by needles.” “I know that you can get treatment. So, if you have it, you can just have a normal life. I have a sister-in-law that is HIV positive. But you have a normal life, she just made a baby. So, she's married. They're having a nice marriage.”

P-2: “I think you can also get it…I am a bit mistaken. Of course, if from a pool or urine or if you touch someone else's blood or something.” “Um…just blood? I imagine maybe semen, but not sure. Not sure if it's true or not. I have no idea.”

P-3: “I would say limited, like I definitely haven't done enough research on it. Um, there is sexually…being infected with blood of someone else who has it? I think that's all the ones I know.” “I don’t particularly know [what fluids transmit HIV].”

P-4: “So, I would say I have quite a good bit of knowledge about it. My dad was a medical doctor. […]. Well, I would say it's transmitted mostly through unprotected sexual contact and also through blood. And then also like through, let's say, injections that are maybe like drug users sometimes use the same syringes and stuff. And I also know that if proper medication is taken like mothers won’t transmit it to their children.” “I would say, well, it's blood, it's semen.”

P-4: “Well, this one [AHF], but other than this… I remember seeing some websites where you can also like order online. Like some of these rapid testing kits, but then that's pretty much it. “

P-5: “I mean not, not too much.” “So, basically either sexual or some blood exchange.”

P-6: “It's a virus and it's confirmed with blood. So, it's…destroy your body after if you don't get medical attention.” “I not sure [how HIV is transmitted].”

P-7: “In the last two days, a lot. Because I researched a lot, yeah. But knowledge? Yeah, I just, I just googled the symptoms. Basically, I know what the symptoms look like and but apart from that medication and stuff I didn't go into that.” “Yeah, by sex. And by…especially if there's some kind of contact between an open wound and yeah with bloods and stuff.” “Yeah, I think only blood [transmits HIV].”

P-8: “I know that it is like the first step towards um, so like the first, so if you don't treat it or get it under control, it can evolve into AIDS.” “Sexually. I think that’s it.” “Sperm for sure and that’s all I know.”

P-9: “It's a virus. I'll say a deadly virus, yea.” “Sex. Mainly by blood transfusion. Yeah, through blood.” “Um, I think that’s it.”

P-10: “[Well, that it’s a virus that is spread by either sexual contact, also by transfusions…blood. Well, also through drug use, they inject themselves or that the needles have been used by other people. Pretty much that.](https://1drv.ms/u/s!AOr7ibsM-DSLgfJC)” “[By means of injection, through unsafe sex, through sores that exist inside the body and that there is a high viral load in some of the fluids.](https://1drv.ms/u/s!AOr7ibsM-DSLgfJC)” “[Um, blood, semen. Not sure, but maybe saliva.](https://1drv.ms/u/s!AOr7ibsM-DSLgfJC)”

P-11: “Well, my knowledge is to be protective in terms of when having sexual intercourse, but I don't have in-depth knowledge as in doctor’s knowledge on HIV.” “Sexually, you have sexual intercourse. You know, without protection of course and someone is infected…either the person is…it's not HIV yet, but it can be. It can be developed into HIV.”

P-12: “A little bit. I don't know much, but I know a little.” “[Transmitted] by sex and by blood.” “Sperm and blood.”

[Window period] “[As I know you have to wait. I know that you have to wait three months or something like this.](https://1drv.ms/u/s!AOr7ibsM-DSLgfJ6)”

**Attitudes towards HIV testing**

P-1: “I think testing is good. I mean, for your own safety, if you have unprotected sex, you should get tested, but even if you don't, it's better to just get the test at least once a year, or at least once at six months. I don't have a problem. I came here. I came first and then I told them (my cousins) and they say, ‘oh, I want to go too’. I say ‘OK. Let's go and I take you’.”

P-2: “Well, I do think it should be more normalized because especially being from Eastern Europe where a lot of the country is not, well, a lot of the countries actually are not that educated in terms of sexual education.” “I do think we should normalize it. It's at the end of the day just the disease, which you can also just fix, which I just learned right now.”

P-3: “I think it's a great thing to have to test for such a thing, because the earlier you catch it, the better your chances are at like dealing with it better or like not letting it, um, grow too much. So, I think it's an effective tool and I think everybody should get tested. It's not exclusive to just one group or another, everybody should.”

P-4: “[I think it's kind of like one of those things that […] people have sex and a lot of people might not know that they might be HIV positive. So, if there was like a culture which advocates for a more repeated testing of HIV, then it prevents it from leading into AIDS and then potentially creating really big consequences for people.](https://1drv.ms/u/s!AOr7ibsM-DSLgfFz)”

P-5: “I mean, yea, it is I think important but I don’t think really too much about it. Only because yea, I don’t know, I thought ok, I don’t need test so, yea.”

P-6: “I think it's important, yea, because if you got it, you can get pills and you can live normal. But if you don't know and you don't test then your body will destroy you. That's what I think.”

P-7: “For me not this important, but I can imagine for a lot of other people, and yeah, maybe gay community it is very important. I had never thought about testing for HIV before.”

P-8: “I think that it can be very like helpful for people that may have it to get it under control and to prevent it from spreading.”

P-9: “I think it's very important for everybody. You know you need to get checked like every time.”

P-10: “[That it is necessary to rule out any situation... to not have any risk and not to spread without knowing to...to other people. I think it is important that they take the test to rule out any situation and if positive, good also to make it known to sexual partners and receive retroviral treatment.](https://1drv.ms/u/s!AOr7ibsM-DSLgfJC)”

P-11: “It is important for people to do it because then you're also aware of whether you're transmitting or not to other potential candidates, so it's important to be safe at any time, not only for yourself, but also towards others.”

P-12: “I think it's very good to test, and I think it's good for everyone to test. I think it's very important to test, but I never had a test. Myself, I've never had an HIV test. I never did it.”

**Social Norms on HIV and Testing**

Taboo and Stigma:

P-1: “Yeah, you know migrants when they come here, they come to work. You know from different parts of the globe from where they come, they have a bad life at home. They don't earn a lot of money. So, they come here for work. They're poorly paid, most of them. Because this is it is what it is so having a free test…knowing that they have a free testing, they would come.” “And in, Romania, for example, this is still taboo. So, this is kind of talking about this, but it's still taboo.” “They maybe they do a little bit of education, but it's…they don't put like an accent, you know? You put the finger on this subject. They don't do it. They just HIV. You do it on school a little bit, but we in Romania, we don't have sexual education.”

P-2: “Well, I do think it should be more normalized because especially being from Eastern Europe where a lot of the country is not, well, a lot of the countries actually are not that educated in terms of sexual education.”

P-4: “The education system does mostly talk about the illness, but with regards to sex, it's like mostly to abstain from it. So, people are aware of it. They're also like free testing. […]. But yeah, it's like there's a bit of stigma with it.”

P-5: “Taboo really. I think the culture [India] says you know these things about HIV - it’s not ok and maybe yea people have fear, I don’t know.”

P-7: “…if I would have HIV, I would not talk about it… because you will be labeled in some kind of way. And that's what I would be afraid of.”

P-11: “[I think looking at myself, that's [taboo] one of the barriers I had to go through myself - living in the Netherlands - because then you see also the difference is in terms of back home, we barely talk about sex and sexuality and sexually transmitted diseases.](https://1drv.ms/u/s!AOr7ibsM-DSLgfJv)”

P-12: “[In Morocco], they can handle it more if you say it's transferred from blood than it's transferred through sex because sex is a taboo.”

K-2: “And the farther we come in our HIV epidemic it will be harder, and I think it's even more structural barriers to whatever - vulnerability, stigma, the care. And it will be even harder to reach these few. For me, the answer is more in making stronger the structural... first like...But it's kind of impossible at this moment, I have a feeling.”

K-4: “Well, I remember one time I was at this health market in Zuid Oost and there would be also people measuring blood pressure and everything, but on the market it's really hard for people to pass by. I mean, everyone sees each other. It's a bit taboo. If you go to the GGD where they have STI and HIV testing, people pass by because there is a taboo. […] But, like the testing - if you do the testing and it's in the school building for instance one afternoon and it's very visible that it is STI testing, people don't go there.”

K-6: “[Among migrants there are many married men who are considered as hetero but have sex with other men and do not identify themselves as gay, bisexual or even MSM due to stigma and taboo. Therefore, they never receive the information they need, the test, and prevention services.”](https://1drv.ms/u/s!AOr7ibsM-DSLgfMx)

Cultural Perceptions of HIV:

P-1: “They talk about sex, but once people you say something about HIV because I told you knowledge and research, they don't do a lot of them and they're afraid like, don't eat with the same spoon.”

P-3: “People were mocking it [HIV testing], they weren't taking it seriously. They thought it was useless, so I think it's it is a problem of availability of testing, but I think it's even a bigger problem about people's portrayal of HIV because then grows a stigma that is hard to break off.” “You can't mention HIV because it's almost like shame. They associate it with ‘Oh, if you're talking about it, you must be doing it.’” “Maybe one is that they [migrants] don't feel like it's necessary, like they're just like, I just don't want to. And two, there are also cases where people are scared.”

P-4: “For example, with the case of Iran, this big emphasis of the education system in letting people know that they need to get [HIV] tested and kind of like these destigmatizing is somewhat recent, so this has happened only in the last 15 years. So, before that it was pretty limited. People just learned from their friends. And with families, it's really coming from a conservative background.”

P-5: “Nowadays it’s quite open compared to the 10 year or 15 years ago. And it's a little acceptable because it's not only a thing that always happened by sexual activity, because it's possible even if you have some blood exchange or some other incident at barber, for example, while doing the shaving. So, people think it's not always the one way of the transfer of HIV.”

P-10: “I think from my generation down it's like you talk more often, like you can talk about HIV without any problem, but with older generations you treat it more delicate because maybe the reaction they're going to have to this is negative.”

Cultural Norms on health:

P-2: “In the big city it's very easy to go to the doctor, but we just have that mentality of like…Because we drink, everyone drinks, everyone smokes, everyone eats a lot of processed meat. Everyone, we are very unhealthy compared to the West, but back home, everything. And that's normal. Like, of course, you're gonna live. That’s the culture.” “Later, more adult. Heterosexual males…It's impossible to convince them to go to the doctor. It’s like, I mean, ‘I'm already 60 I’m going to die soon. I feel fine, yeah. Like I already had this pain for three years. Yeah, it is what it is.’”

P-12: “And I think also, that's something you would not find openly in Morocco, because, of course, it's a Muslim country. There are many NGO's who do it, who provide condoms etcetera. But with the campaign they will not openly talk about providing condoms because then you're saying you're stimulating sex.”

Expectations of Care:

P-2: “F[or example, back home, if I register to my GP, I don't have to be in that area. I can just go to one in another city](https://1drv.ms/u/s!AOr7ibsM-DSLgfFq).” “Well, that there is friendly people to help you and make you relax. I hope they have the right knowledge, and you know, to explain me everything.”

P-5: “Really, I just want to make sure that ok, that on that day something that happened I was not aware…How I can make sure that and make, maybe we can say confusion-free because I'm confused whether I have something or not. So, there's like a confirmation.”

P-6: “I thought for the yeah, I thought I come here and I test and I yeah, I get them in the 30 minutes - says on the Internet, the results. OK, so yeah, that was it.”

P-8: “Honestly, I had good expectations just because I read the [AHF] website and I was like, this sounds like a very welcoming place, a safe space and so far it has been like that.”

P-9: “The process of the test was good and easy.”

P-12: “That I feel that they would give me a feeling of comfort. I think that's very important. That be comfortable and that it will be between us or something like that, very confident.”

K-1: “[But still their [migrants] expectancy from the healthcare might be quite different because of their cultural background. So, they go to a doctor, and they expect to get some medication whilst in the Netherlands; GPs are there to kind of advise and counsel. They [migrants] are also disappointed in what they get.](https://1drv.ms/u/s!AOr7ibsM-DSLgfMx)”

K-5: “People from different cultural backgrounds, they all have some trust issues with GPs because they seem to think the GP only gives them paracetamol, and advises them to come back in two weeks.”

K-6: “Depending on the place they live they can go to the healthcare center in the asylum seekers center or go to their family doctor. At the beginning the migrants are totally unaware of the healthcare system in the Netherlands and the GP is their only contact point with the health system.”

K-7: “One of the things that really is an issue, is not only the bureaucracy of it all, but also the comment: ‘Start by taking paracetamol.’ This can be a culture shock and can result in care avoiders in the future.”

**Perceived Behavioral Control over HIV**

P-1: “Yeah, I was pretty much careful. Like pretty much careful in the past. That's why I was, like, freaking out. Because what happened with this guy was just like a mistake and it freaked me out. But yeah, I'm careful. That's why I didn't get tested. I had a stable relationship.” “If I if we do sex or like this with someone that I don't know after my relationship. I always use protection. Like condoms.”

P-2: “I was afraid. I think fright is by far the biggest concern I had because I'm afraid of a lot of stuff and like I am hypochondriac.”

P-3: “I just never thought about it for a while until I first tested and um…Yeah, I've never had that sentiment of, like, I don't want to get tested because it feels like just any other medical test that you would want to know if you have a certain thing or not.”

P-4: “Well, if let's say I'm going into like a new relationship that I don't know the let's say the HIV history of the other person or better, I've had like let's say like a one in stand or something with some other people and then I'm going into relationship and then want to make sure that OK, I'm not transmitting it then that is kind of like a big incentive to get tested.”

P-5: “Yeah. So, basically almost two months ago I had some party. And I was drunk almost, and I don't know what happened that night.” “Actually, after that I just came to the NL. Two weeks so, that's the reason - I was on holiday…um when the party happened, when I was drunk. But there is no such reason like I have some social pressure or something like that. That's not a reason.”

P-6: “My doctor says it's not necessary because it's very low risk with Dutch girls. Only if I slept with [girls from] other countries. He told me that. He says not necessary for me now, because you don’t sleep with [girls] in the Red [Light] districts.”

P-7: “I've had the same sex partner for four months, but I know that she has been in Latin America. I know what she did there but there might be a chance that you know…and I had unprotected sex with her because she's like my fixed partner, you know?

P-8: “Because I was using protection, like condoms.”

P-10: “[I hadn't done it before because well, um, I spent more than 5 years without a sexual partner, so it was like there was no need because I didn't use drugs that were injectable nor did I hire services to have sex and... nothing, I had never received transfusions, so it was like ‘what for’? It was like ‘I don't need it right now because nothing is happening right now.’ Now I have a sexual partner and ok, we have an open relationship and it's like I say, OK, let's get tested to avoid any risky situation.](https://1drv.ms/u/s!AOr7ibsM-DSLgfJC)”

P-11: “[If you know somebody for a long time and you had a long-term relationship, then there's no need to test.](https://1drv.ms/u/s!AOr7ibsM-DSLgfJv)” “I didn’t test for HIV since I believe I was always safe.”

P-12: “I really don't know why…maybe because I never felt, how do you say, maybe I never felt vulnerable to test.”

K-6: “I think it depends strongly on their country of origin and on the HIV epidemic in their country. People who come from specific African countries where they have experienced the worst aspects of the HIV epidemic fear, are not necessarily familiar with the fact that with a good treatment they can live as long as people without HIV. Therefore, they don’t want to get tested and deny the risks.”

**Availability of HIV Testing Services**

P-3: “[I only know of this one [AHF Checkpoint] and I know that they go to Rotterdam every Wednesday, so it's the same organization. They have other locations. Sometimes, I know that they went to Eindhoven a few times.](https://1drv.ms/u/s!AOr7ibsM-DSLgfIo) [I don't know of any other organization that does the same.](https://1drv.ms/u/s!AOr7ibsM-DSLgfIo)”

P-2: “Yeah, I was super lucky with this. So, my company brought me here from Romania and they had a software that they collaborated with, and that software is like your personal butler, let's say for a couple of months to help you settle in and they gave you all the information like where and how you can find a house, or how you should register to a GP.”

P-9: “[I feel like this is the easiest place [AHF Checkpoint] because I came here because I couldn't find anywhere open today [Saturday]. Or maybe you have to like book appointments, so it's really convenient and very quick for me.”](https://1drv.ms/u/s!AOr7ibsM-DSLgfIz)

**Accessibility to HIV Testing Services**

P-5: “[And, basically with the challenge of where I can go without appointment quickly […], because making appointment is very difficult here in Amsterdam.](https://1drv.ms/u/s!AOr7ibsM-DSLgfF2)”

P-6: “[It's difficult because it's not very easy…places to find with information [on HIV testing]. Yeah, nobody talks about this. I read many people don't know they got HIV here in the Netherlands, so they live with HIV and they give to all the others because they don't know they got it.](https://1drv.ms/u/s!AOr7ibsM-DSLgfIs)”

P-7: “[It has been pretty bad actually with the GGD because I remember that I wanted to [test] there because friends told me about that you could get free STD test until your 25^th^. After your 25^th^ you have to start paying for it.](https://1drv.ms/u/s!AOr7ibsM-DSLgfIx)”

P-10: “[For example, if I am here, because I live in Haarlem. But if I want to search in the GGD of Amsterdam it’s like at the time of putting in the post code and your post code is from Haarlem, you can do absolutely nothing in Amsterdam, and they close the questionnaire and send you to Haarlem.](https://1drv.ms/u/s!AOr7ibsM-DSLgfJC)”

K-3: [“Actually, we changed our system for making an appointment to an online one. And, by doing it we saw that we were missing persons who were not Dutch-speaking and not online literate enough. But I think the online appointment is an extra handicap because you just cannot phone and make an appointment or ask questions. You have to fill out a form online. And that's too hard. I think even if you're Dutch and not very literate online, that's really a problem.](https://1drv.ms/u/s!AOr7ibsM-DSLgfMx)”

**Openness to Talk about HIV and/or Testing**

P-3: “[I think with my friends it would be fine, but I think if it's with family then it's a bit taboo because there's a lot of misinformation about the way it's transmitted and how it first started and who in particular is it more common with, then all these assumptions. It wouldn't be a good topic to bring up.](https://1drv.ms/u/s!AOr7ibsM-DSLgfIo)”

P-7: “ [I think there's other STD's that are more likely to be brought up during discussion, but not HIV because where I work it’s not a branch where people talk about HIV. Maybe about other STD's. But honestly, I've never talked to my colleagues about it.](https://1drv.ms/u/s!AOr7ibsM-DSLgfIx)”

P-12: “[The topic is no problem, I think - to talk about HIV. But if I have to do a test, then it will be another thing. Then it is ‘did you sleep with somebody else?’, ‘did your husband sleep with somebody else?’, ‘why should you take a test?’ I think that would be if I told somebody from my background.](https://1drv.ms/u/s!AOr7ibsM-DSLgfJ6)”

**Client’s Perceived Need for HIV Test**

P-5: “[I mean, if I'm not doing any sexual interaction with any other partners, and I don't have any drugs addiction. […]. I mean, it's not always it should happen with only sexual intercourse. For example, if we are exchanging some drugs and all the blood exchange or something like this, the injection.](https://1drv.ms/u/s!AOr7ibsM-DSLgfJ6)”

P-7: “Symptoms, and the fact that I had had sex, have sex with a girl that has been in another country for a long time, so also a bit of the other countries involved.”

P-9: “No, I just will go and check if maybe you know there was some exposure or something like that.”

P-10: “I hadn't done it before because I spent more than 5 years without a sexual partner, so it was like there was no need because I didn't use drugs that were injectable nor did I hire services to have sex. I had never received transfusions, so it was like ‘what for?’ It was like ‘I don't need it right now because nothing is happening right now.”

**Evaluated Need for HIV Test**

P-6: “[My doctor says it's not necessary because it's very low risk with Dutch girls. Only if I slept with girls from other countries. He told me that.](https://1drv.ms/u/s!AOr7ibsM-DSLgfIs) [He says not necessary for me now, because you don't sleep with [girls] in the Red [Light] districts, it's just not necessary.](https://1drv.ms/u/s!AOr7ibsM-DSLgfIs)”

P-6: “Then I don't want to do with the testing. Yeah, I go to my doctor then.”

P-3: “Answering all the questions that people might have, because in some cases people have a lot of fear towards this thing [HIV testing]. Even if, like you were positive, that it's fine. It's just like any other virus; you can contain it. But yeah, also providing a safe space.”

P-10: “I think the fact that I don't feel judged. Let me know that I am in a safe place. That they are not going to make any judgment or something that could affect me negatively.”

K-7: “Healthcare professionals have multiple reasons not to test for HIV. For example, they don’t want to come across as stigmatizing because their client is from a certain country. But sometimes healthcare professionals don’t test for HIV because their client isn’t MSM.”

**Use of Health Services (i.e., HIV Testing Services)**

P-3: “[Answering all the questions that people might have, because in some cases people have a lot of fear towards this thing [HIV testing]. Even if, like you were positive, that it's fine. It's just like any other virus; you can contain it. But yeah, also providing a safe space.](https://1drv.ms/u/s!AOr7ibsM-DSLgfIo)”

P-4: “Pretty good. Like, I think most people who are working in such places [AHF], but I think they have a pretty good understanding of HIV. So, they are also aware of the stigmas that are related to it in some people.”

P-6: “[Then I don't want to do with the testing.](https://1drv.ms/u/s!AOr7ibsM-DSLgfIs) [Yeah, I go to my doctor then.](https://1drv.ms/u/s!AOr7ibsM-DSLgfIs)”

P-6: “Yes, safety feeling. So, if you got it [HIV], it's not so bad. You get pills and safety talk.”

P-7: “There's a difference because here [AHF] you walk in, you get a test, they show you a bit what's going to be like and the other HIV test is with my doctor and I'm telling OK, this is what's wrong with me. And then she tells me, OK, you can do a blood test, you can come here tomorrow at 8:00 at this particular space, they will take blood and we will do the HIV test. And when you come the next morning, there's a completely different person who's going to take your blood. She probably has no idea what your blood is going to be used for because she's using an app and making screenshots and so it's less personal.”

P-9: “[It's much different from here [the Netherlands], you know, even just going for an HIV test. The eyes you get from people around even coming up the stairs to this place [AHF], like people in the store downstairs, they be looking like ‘where you going?’ They be staring, you know like ‘what you doing?](https://1drv.ms/u/s!AOr7ibsM-DSLgfIz)’”

P-9: “Yeah, I mean, you get very scared because if you're like positive, I mean it's a scary thing, even though, like, OK yeah, it's manageable, but I feel like it's very scary just to know you're like, positive. So, that's how I think everyone feels before the test. They feel scared and they want like a quick result. You want to know where you stand.”

P-10: “[I think the fact that I don't feel judged. Let me know that I am in a safe place. That they are not going to make any judgment or something that could affect me negatively.](https://1drv.ms/u/s!AOr7ibsM-DSLgfJC)”

P-11: “[If that person is open and able to have good conversation skills and be able to relax with you. Help to have a good talk, a good chat…those are all the attributes that add up.](https://1drv.ms/u/s!AOr7ibsM-DSLgfJv)”

**Dutch Healthcare System**

P-2: “[…]. And I don't think anyone cares. Like when I say Romania, like when I’m eating...they don't care, so that was very amazing. And same in hospitals. I went to the doctor and I said, well, I'm from Romania. OK. You're not gonna ask me anything? So, no, no discrimination.”

P-6: “[I know this place [AHF Checkpoint] yeah, and [the clinic] in Utrecht - it's fast test, 14 days [after risk] it says, and they want €300 euro. But I don't do that, too much money.](https://1drv.ms/u/s!AOr7ibsM-DSLgfIs)”

P-10: “S[o, we already knew it was going to be like ok, let's review what options the GGD offers and after GGD it’s like, ok well, we saw that the GGD does not work, let's look for other options](https://1drv.ms/u/s!AOr7ibsM-DSLgfJC).”

P-10: “No, I have not felt any discrimination in the health sector. I have received attention in everything without any problem. Maybe I've been more in touch lately with doctors and that communication has now been in Dutch. And sometimes I have a hard time pronouncing a few words, but that's okay. I just continue the conversation in English, but I've never felt like 'hey, you have to talk Dutch...' or some discrimination, like ‘Ah you are an expat’ or for anything else, never. Not here, no.”

P-12: “Yeah, I only know that you have…we always say in the Dutch - you have to exaggerate your pain […] to get the really good advice or referral from the doctor. I know this. I always say that also to other people. If you don't speak the language [Dutch] very good, you don't speak their language…they don't take you very serious.”

K-1: “[But it's not a governmental task. That's exactly the point. That's the decentralized system we have in the Netherlands and that means that this task is at the GGDs. They are the ones that have to address preventive measures towards the people within their region and they can issue leaflets or whatever in any language you want if you have the finances for that](https://1drv.ms/u/s!AOr7ibsM-DSLgfMx).”

K-1: “But it's a complicated system […], it's not clear what is paid for, what is not, […] who has access to it.”

K-2: “I think health literacy in general is the biggest problem because they [migrants] don’t know where to go or have the knowledge that it [HIV testing] might be useful.”

**Influence of External Environment on the Use of HIV Testing Services (External Environment)**

P-10: “But at the GGD if you don't meet certain requirements, it's like ‘Ah, no, you're not a candidate.’ Yes, my partner and I checked yesterday and it was like 'Oh don't you meet these requirements? well, you better go first to be checked by the doctor and from there they can refer you.’”

P-10: “[…] you start doing the questionnaire and after the third question, they [GGD] ask to put your post code. It's like, OK, you put it in and it closes and says, ‘Ah, you can't advance any further,’ the questionnaire closes and you're not a candidate to go to the ones here in Amsterdam. ”

K-1: “There's hardly anything there because that’s not the policy of the ministry. […] We do want to address for instance condom use, so we want to make a campaign that's broader for a lot of the public. Advertising the use of condoms. But they are absolutely directed towards the general public. There is no emphasis towards migrants at all.”

K-1: [“They [GGD] will prioritize those who are warned for an STI or who have symptoms of an STI. So, just being young or just being from an endemic country doesn't give you access](https://1drv.ms/u/s!AOr7ibsM-DSLgfMx).”

K-3: “And that's why we are always very happy that we have the finance only partly from the municipality and the larger part is governmental.”

K-3: “But even when we have within the limited PrEP room space, we got a room, I mean like 2000 people. We had the policy to make separate room for vulnerable non MSM population but it didn't get filled up so we tried to give more information at refugee center about PrEP but we have room for more vulnerable persons, but they didn't come.”

K-4: “[But they [migrants] don't have to show proof. So, I mean they [SHC] can never check if they're really living there. So, you can call to Haarlem, and you say you're from Haarlem and you might be accepted at the Public Health Service](https://1drv.ms/u/s!AOr7ibsM-DSLgfMx) [SHC], and they won’t ask for proof.” […] “[No, they don't know.](https://1drv.ms/u/s!AOr7ibsM-DSLgfMx)”

**HT Participant’s Perception of Overall Health** **(Perceived Health Status)**

P-3: “I think the one reason that would make me want to test is if I developed symptoms that are similar or akin to HIV. So, I think for me it's good to develop knowledge about that at least. Because yeah, it would be helpful.”

P-4: “Well, if, let's say, I'm going into like a new relationship that, I don't know the HIV history of the other person or better, I've had like a one-night stand or something with some other people and then I'm going into relationship and then to make sure that OK, I'm not transmitting it then that is kind of like a big incentive to get tested.”

P-9: “[Yeah sometimes you know, you have maybe a flu, and it takes longer to go away and it’s like, ‘why is it taking so long?’ You know, stuff like that. But mainly it's personally just because of sex.](https://1drv.ms/u/s!AOr7ibsM-DSLgfJ6)”

P-12: “[If I have symptoms, I don't know if I think about HIV. It's not the first thing I think ‘Oh, maybe I have HIV. If I had headache or I think my blood pressure is low then I will go to the doctor, but I wouldn't think about HIV at this moment](https://1drv.ms/u/s!AOr7ibsM-DSLgfJ6).”

**HT Participant’s Health being Assessed by Health Professional at Medical Facility (Evaluated Health Status)**

P-7: “[Yeah, but I don't go there with the idea I want to do an HIV test. I just go there with things that I feel maybe are bad and then they said, ‘you know, just to be sure, let's do an HIV test. It’s not that I went there because I wanted to take one. Since I was already there they brought it up because of the symptoms I was having, to make sure.](https://1drv.ms/u/s!AOr7ibsM-DSLgfIx)”

K-2: “Soa Aids Nederland has been trying to train GPs, especially in Amsterdam but also outside, in diagnostic testing, which is about you're not going to find HIV, you're going to be sure there is no HIV.”

K-5: “[So, in the general GP training, there's no HIV specific curriculum, but we of course have training in STDs but it's pretty superficial. As GP’s we try to assess ‘risk behavior’ and give our patients some education about sexual behavior and its risks. STD diagnostics are offered according to (risky) behavior and/or patients’ wishes. However, we do not outreach to all our patients.”](https://1drv.ms/u/s!AOr7ibsM-DSLgfMx)

K-7: “There is a lack of testing for HIV even when there is an indicator condition. Healthcare professionals have multiple reasons not to test for HIV. For example, they don’t want to come across as stigmatizing because their patient/client is from a certain country.”

**Suggestions from HT participants and Key Informants**

P-4: “…they [the Netherlands] need more education specifically on HIV.”

P-5: “I mean, I don’t know…maybe more places with no appointment for test.”

P-6: “I don't know, I think, yeah, maybe the place where they stay in AZC. Maybe they need to give information.”

P-7: “Well, I think the best thing to do in these times is to be visible online. So, if I type into my safari or Google Chrome Free HIV test Amsterdam, you guys [AHF] need to be top ranked. That's the way people find things nowadays. That's the way I would do it if I were in a different country as well, and I needed a test.”

P-8: “Maybe if this topic about [HIV] testing was more talked about in different institutions. Like at university for example.”

P-9: “Availability. Like having places be open on Saturday, maybe like this.”

P-10: “[I do not feel that there is as much promotion of 'come get tested’ or ‘come to these places’. I mean, I see more things like, I don't know, do your taxes, you know, things like this.](https://1drv.ms/u/s!AOr7ibsM-DSLgfJC) [It’s not like we enter the country and the first thing they tell you is ah, you have just arrived, take all your things, this is your welcome kit, no.](https://1drv.ms/u/s!AOr7ibsM-DSLgfJC)”

P-12: “Education. Also, a lot of [target] groups are in the neighborhood - where you come, where you live – there has to be more information there.”

K-1: “The GP is there for people who expect they have a problem, but a lot of migrants wouldn’t know they had risk behavior or that they are at risk of contracting HIV. […] I mean, for somebody who's entering the Netherlands as an adult, there is not a single moment in their life that they will be getting any information apart from the Internet but also then you will have to have an incentive to be looking for information, and if you don't have that incentive then you're lost.”

K-6: “Tailored projects for each sub-population based on their background, information level and sexual behavior. The projects should include awareness raising and making low-threshold services available for everybody.”

K-6 “Giving information about test and treatment possibilities. Normalizing test, offering test with other routine tests.”

K-7: “Recognizability. Making sure that the way organizations communicate about HIV (testing) is relatable to the people you are trying to reach. The fact that we see such a high percentage of late presentation in this group also supports the claim that from a public health perspective, testing for HIV should be free of charge. This way we also don’t disproportionately burden the people who have less financial means.”
